# Supplementary material for: Characterization of tigurilysin, a novel human CD59-specific cholesterol-dependent cytolysin, reveals a role for host specificity in augmenting toxin activity
Source: Microbiology (Reading). 2023 Sep 13;169(9):001393. doi: 10.1099/mic.0.001393 (PMC10569062; doi:10.1099/mic.0.001393)
Supplement: Supplementary material 1 [file mic-169-1393-s001.pdf]

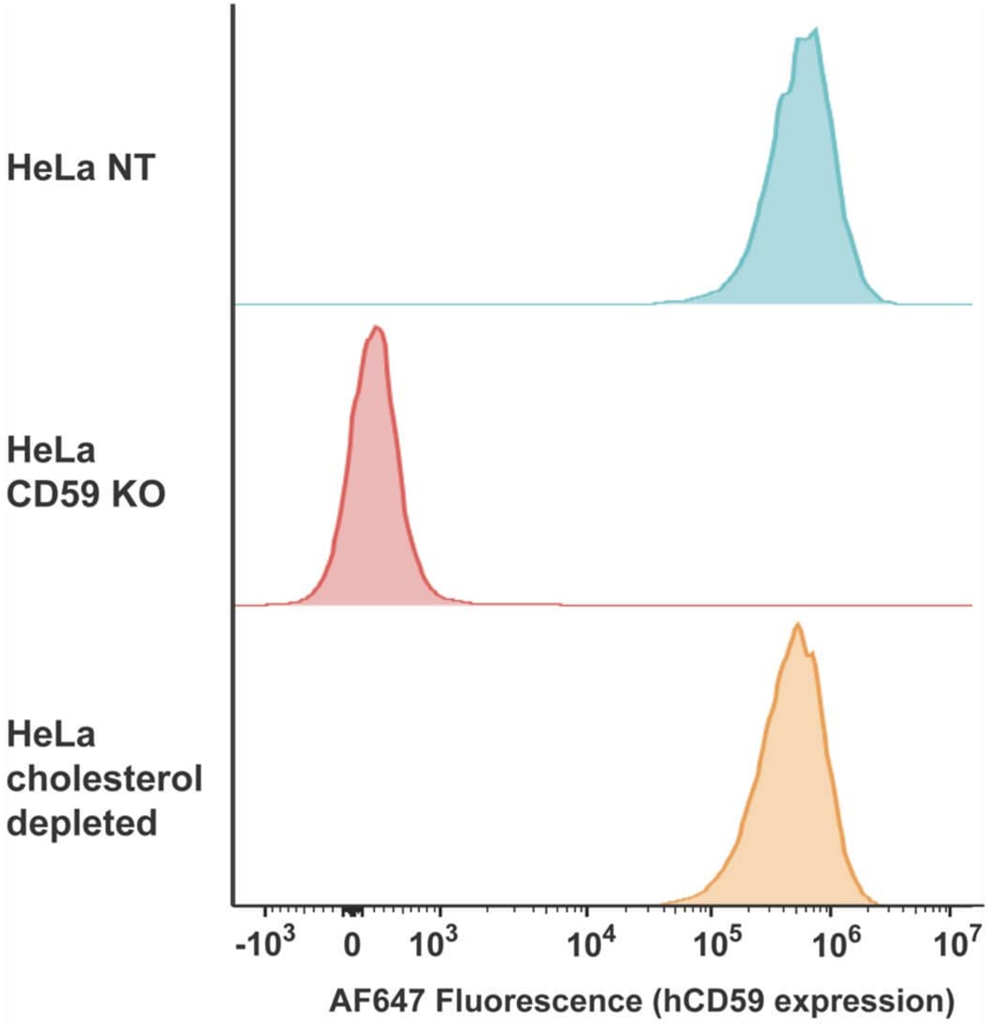

Figure S1: Cholesterol depletion does not affect hCD59 expression on HeLa cell surface
Flow cytometry analysis of hCD59 expression on the cell surfaces of HeLa cells (NT control, hCD59 KO, and cholesterol depleted) using an anti-hCD59 primary antibody and an Alexa-Fluor 647 (AF647) secondary antibody. Histogram x-axis shows APC fluorescence intensity of the secondary antibody for each cell population. Histogram y-axis shows cell count normalized to mode.

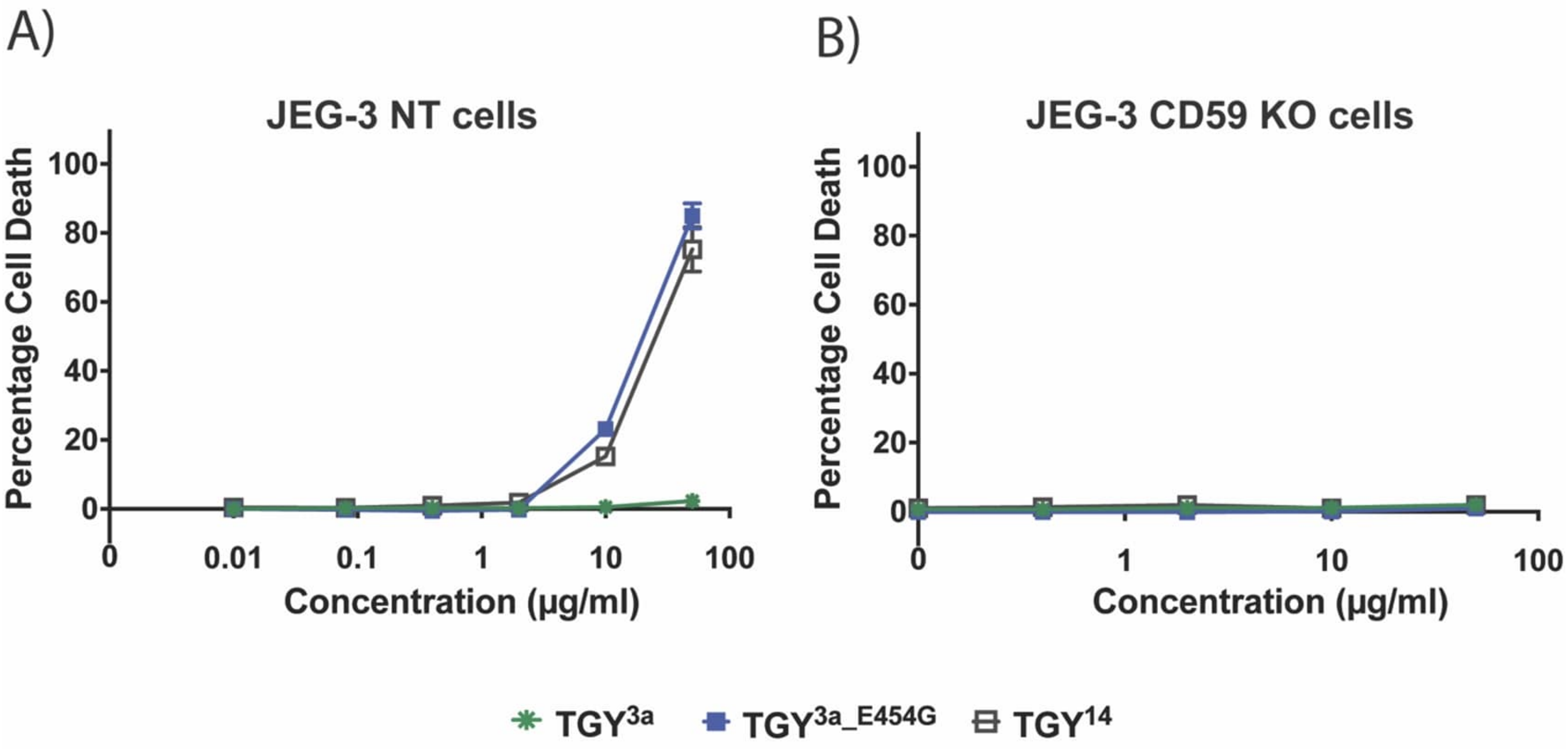

Figure S2: Functional TGY variants lyse JEG-3 cells in a hCD59-dependent manner

(**A-B**) Percentage cell death in JEG-3 NT control cells and JEG-3 hCD59 KO cells when exposed to increasing concentrations of the three TGY variants. Cells were incubated with toxins for 1.5 hours and cell death measured by an LDH-release cytotoxicity assay. Each point is the mean of 3 replicates, and error bars represent ±SD.

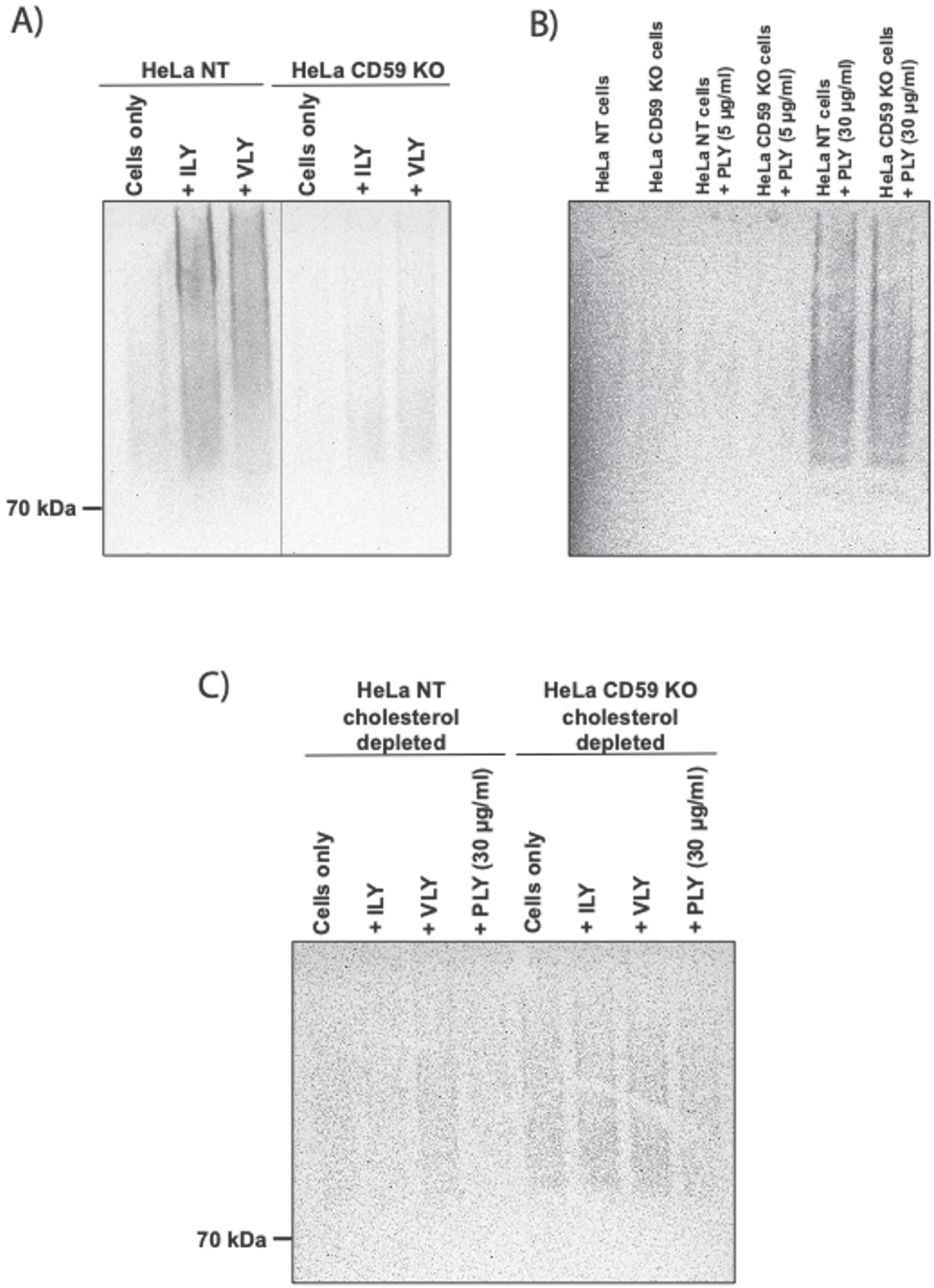

Figure S3: Visualizing CDC oligomers for ILY, VLY and PLY on HeLa cells

(**A-C**) Oligomerization of CDCs as assessed by SDS-AGE analysis. All CDCs were used at a concentration of 5 µg/ml, unless indicated otherwise. Cells were incubated with the indicated toxins on ice for 10 minutes before being prepared for western blotting. 0.01% glutaraldehyde was used as a cross-linker to preserve oligomers during sample preparation. Western blot membrane was probed with an anti-His-tag HRP-conjugated antibody.

	Primer name	Sequence (5'-3')
1.	PLY_D1-3_F	CTGGTGCCGCGCGGCAGCCATATGGCAAATAAAGCAGTAAATG
2.	PLY_D1-3_R_tgy	CGTTTTTATAAGCTGTAACCTTAGTCTC
3.	TGY_D4_F_ply	GGTTACAGCTTATAAAAACGGCTACCTG
4.	TGY_D4_R	AGTGGTGGTGGTGGTGGTGCTTAGTTGTTTTCAATTTCTTCG
5.	TGY_E454G_F	CACAAGGGTGGTTATGTGGCGC
6.	TGY_E454G_R	ATGCAGATTCAGGTAGCC
7.	TGY_E454A_F	CACAAGGGTGCATATGTGGCG
8.	TGY_E454A_R	ATGCAGATTCAGGTAGCC
9.	TGY_I544L_F	CGGCACCACCCTGCGCCCGAAAT
10.	TGY_I544L_R	TAGTTGGTGATGGTGCGTTTTTG
11.	T7_F	TAATACGACTCACTATAGGG
12.	T7_Term	GCTAGTTATTGCTCAGCGG

Table S1: Primers and oligonucleotides

Primers and oligonucleotides used for construction of hybrids (primers 1-4), site-directed mutagenesis (primers 5-10), and sequencing reactions (primers 11-12).
